# Double Trouble: A Diagnostic Challenge of Coexisting Encapsulating Peritonitis and Transverse Mesocolic Internal Hernia Presenting As Acute Small Bowel Obstruction

**DOI:** 10.7759/cureus.115192

**Published:** 2026-08-25

**Authors:** Jyoti Choudhary, Deepshikha Arora, Chandra Kishore, Avinash Dhok, Mohd Yunus Shah

**Affiliations:** 1 Radiodiagnosis, All India Institute of Medical Sciences (AIIMS), Nagpur, IND; 2 Radiodiagnosis and Interventional Radiology, All India Institute of Medical Sciences (AIIMS), Nagpur, IND; 3 Department of Surgery, NKP Salve Institute of Medical Sciences and Research Centre, Nagpur, IND

**Keywords:** abdominal cocoon syndrome, computed tomography, encapsulating peritonitis, internal hernia, sclerosing encapsulating peritonitis, small bowel obstruction, transmesocolic hernia

## Abstract

Encapsulating peritonitis (EP), also known as abdominal cocoon syndrome, and internal hernia are uncommon yet important causes of small bowel obstruction (SBO). Their true coexistence is exceedingly rare and presents a unique diagnostic challenge. Despite their distinct pathogenesis, both entities can rarely share considerable clinical and radiological overlap.

We report the case of a 29-year-old man who presented with acute abdominal pain and multiple episodes of non-projectile, non-bilious, non-blood-stained vomiting. Contrast-enhanced computed tomography demonstrated clustered dilated small bowel loops enclosed within an enhancing fibro-collagenous membrane, consistent with EP. In addition, mesenteric vascular convergence and a focal transition point within the transverse mesocolon raised suspicion for a concomitant internal hernia. Emergency exploratory laparotomy confirmed a dense fibro-collagenous membrane encasing the small bowel, together with a transmesocolic internal hernia through a defect in the transverse mesocolon. Complete excision of the encapsulating membrane, meticulous adhesiolysis, reduction of the herniated bowel, and closure of the mesocolic defect resulted in complete resolution of the obstruction and an uneventful postoperative recovery.

This case highlights the exceptionally rare coexistence of EP and a transmesocolic internal hernia, two entities that have traditionally been described as radiological mimics rather than concurrent pathologies. Previous case reports have primarily described EP and internal hernia as independent causes of SBO, rarely also highlighting one entity as a radiological mimic of the other; however, their concurrent occurrence in the same patient is extremely rare. This case highlights the importance of a systematic computed tomography evaluation, as recognition of one diagnosis should not preclude careful assessment for the other, with direct implications for surgical planning and optimal patient management.

## Introduction

Small bowel obstruction (SBO) is a common surgical emergency, most frequently resulting from postoperative adhesions, external hernias, and neoplasms. Less common etiologies, including encapsulating peritonitis (EP) and internal hernias, pose a significant diagnostic challenge because of their nonspecific clinical presentation, rare occurrence, and the potential for bowel ischemia if diagnosis is delayed [1-3].

EP, also known as abdominal cocoon syndrome or sclerosing EP, is characterized by partial or complete encasement of the small bowel within a dense fibro-collagenous membrane. It may occur as a primary (idiopathic) condition or secondary to chronic peritoneal inflammation, peritoneal dialysis, abdominal tuberculosis, or previous abdominal surgery. In contrast, internal hernias result from herniation of bowel through congenital or acquired mesenteric defects, with transmesocolic hernias representing a rare but clinically important subtype [1-4].

Contrast-enhanced computed tomography (CECT) plays a pivotal role in the evaluation of SBO and remains the imaging modality of choice for identifying uncommon etiologies. However, EP and internal hernias can demonstrate overlapping CT features, including clustered bowel loops, a sac-like configuration, mesenteric crowding, and proximal bowel dilatation, making accurate preoperative differentiation challenging. Furthermore, the coexistence of these two entities in the same patient is exceedingly rare, and both conditions are reported more commonly in younger individuals than in the elderly population [2,3,5-8].

Most published reports describe EP and internal hernias independently or as differential diagnoses, with one entity mimicking the other; their coexistence in the same patient has been reported only rarely. In contrast, our case demonstrates the simultaneous occurrence of EP and a transmesocolic internal hernia presenting as acute SBO in a young patient [1-5,7,8].

This case highlights the importance of a systematic CECT evaluation for detecting concurrent pathology and highlights that the identification of one diagnosis should not preclude careful assessment for the other, with important implications for surgical planning and patient management. Radiologists and surgeons should maintain a high index of suspicion for these uncommon causes of SBO, particularly in younger patients presenting without a history of prior abdominal surgery or other common predisposing factors.

## Case presentation

A 29-year-old man presented to the emergency department with acute-onset diffuse abdominal pain of two days' duration, accompanied by multiple episodes of non-projectile, non-bilious, non-blood-stained vomiting. He reported recurrent but self-limiting episodes of abdominal pain and vomiting over the preceding six months, with a gradual increase in frequency, suggestive of intermittent bowel obstruction. There was no history of fever, hematemesis, melena, altered bowel habits, jaundice, or urinary complaints.

On presentation, the patient was hemodynamically stable. Physical examination revealed mild abdominal distension with diffuse tenderness, without guarding or rigidity. Laboratory investigations (Table 1), including inflammatory markers and routine biochemical parameters, were within normal limits.

**Table 1:** Baseline laboratory blood investigations at the time of presentation ALP: alkaline phosphatase; ALT: alanine aminotransferase; AST: aspartate aminotransferase; AFP: alpha-fetoprotein; CEA: carcinoembryonic antigen; CA 19-9: carbohydrate antigen 19-9

Blood parameter	Patient’s blood value	Reference range
ALP	108 U/L	<130 U/L
Albumin	2.73 g/dL	3.5-5.2 g/dL
ALT	23.3 U/L	0-45 U/L
AST	17.3 U/L	0-45 U/L
Total protein	5.10 g/dL	6.6-8.7 g/dL
Direct bilirubin	0.214 mg/dL	0-0.3 mg/dL
Blood urea	9.62 mg/dL	15-39 mg/dL
Total bilirubin	0.290 mg/dL	0.2-1.2 mg/dL
Serum creatinine	0.484 mg/dL	0.7-1.3 mg/dL (adult male)/0.5-1.1 mg/dL (adult female)
Serum sodium	134 mmol/L	135-145 mmol/L
Serum potassium	3.7 mmol/L	3.5-5.0 mmol/L
Serum lipase	29.1 U/L	13-60 U/L
Serum amylase	40.5 U/L	28-100 U/L
AFP	8.74 ng/mL	<7.0 ng/mL (adult)
CEA	1.37 µg/L	<3.8 µg/L (non-smoker, 20-69 yr)
CA 19-9	4.69 U/mL	<37 U/mL

**Figure 1:**
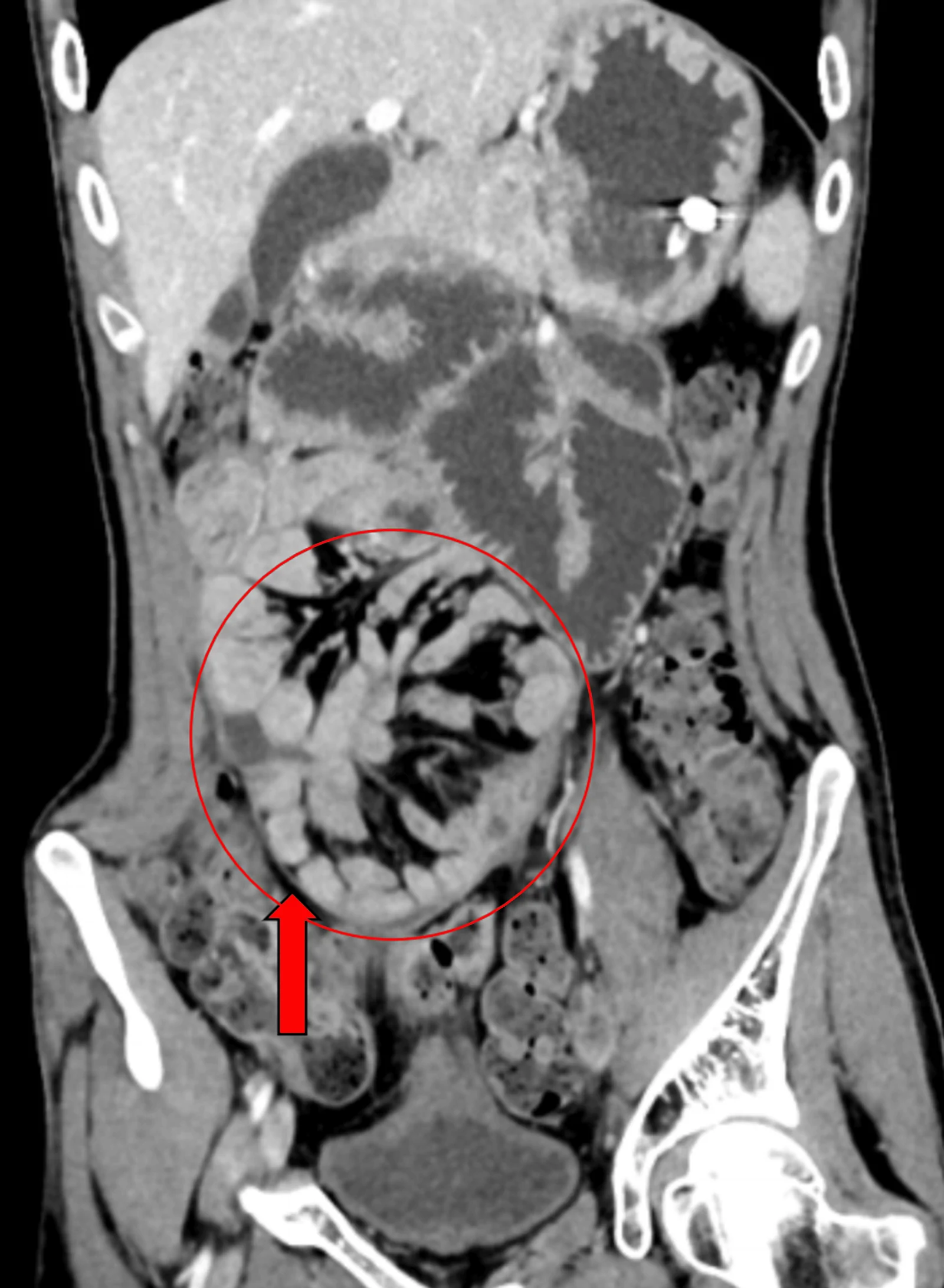
CECT features of EP (abdominal cocoon syndrome) Contrast-enhanced coronal CT image of the patient's abdomen demonstrates a cluster of mildly dilated small bowel loops (red circle) confined to the central abdomen and enclosed by a smooth, enhancing fibro-collagenous membrane (red arrow), producing the characteristic imaging appearance of EP (abdominal cocoon syndrome). CECT: contrast-enhanced computed tomography; CT: computed tomography; EP: encapsulating peritonitis

**Figure 2:**
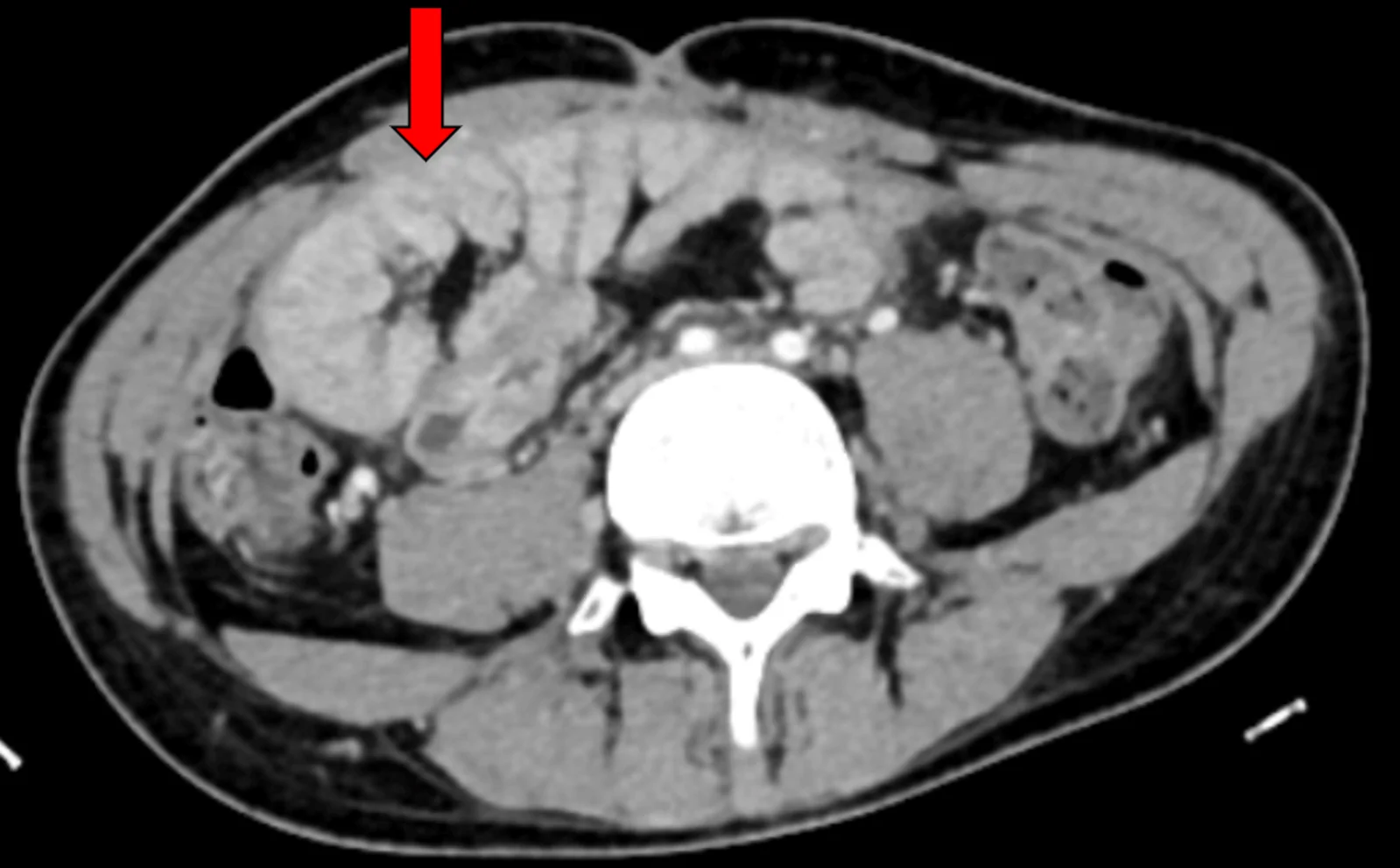
Axial CECT image of the patient demonstrates centrally clustered small bowel loops enclosed by a smooth, enhancing fibro-collagenous membrane (red arrow), characteristic of EP (abdominal cocoon syndrome) CECT: contrast-enhanced computed tomography; EP: encapsulating peritonitis

In view of the clinical suspicion of mechanical SBO, CECT of the abdomen was performed. Imaging demonstrated a cluster of mildly dilated small bowel loops confined to the central abdomen and enveloped by a smooth, enhancing fibro-collagenous membrane, producing the characteristic appearance of EP (abdominal cocoon syndrome) (Figures 1, 2). Mild inter-bowel fluid, tethering of adjacent bowel loops and mesenteric crowding were also evident.

**Figure 3:**
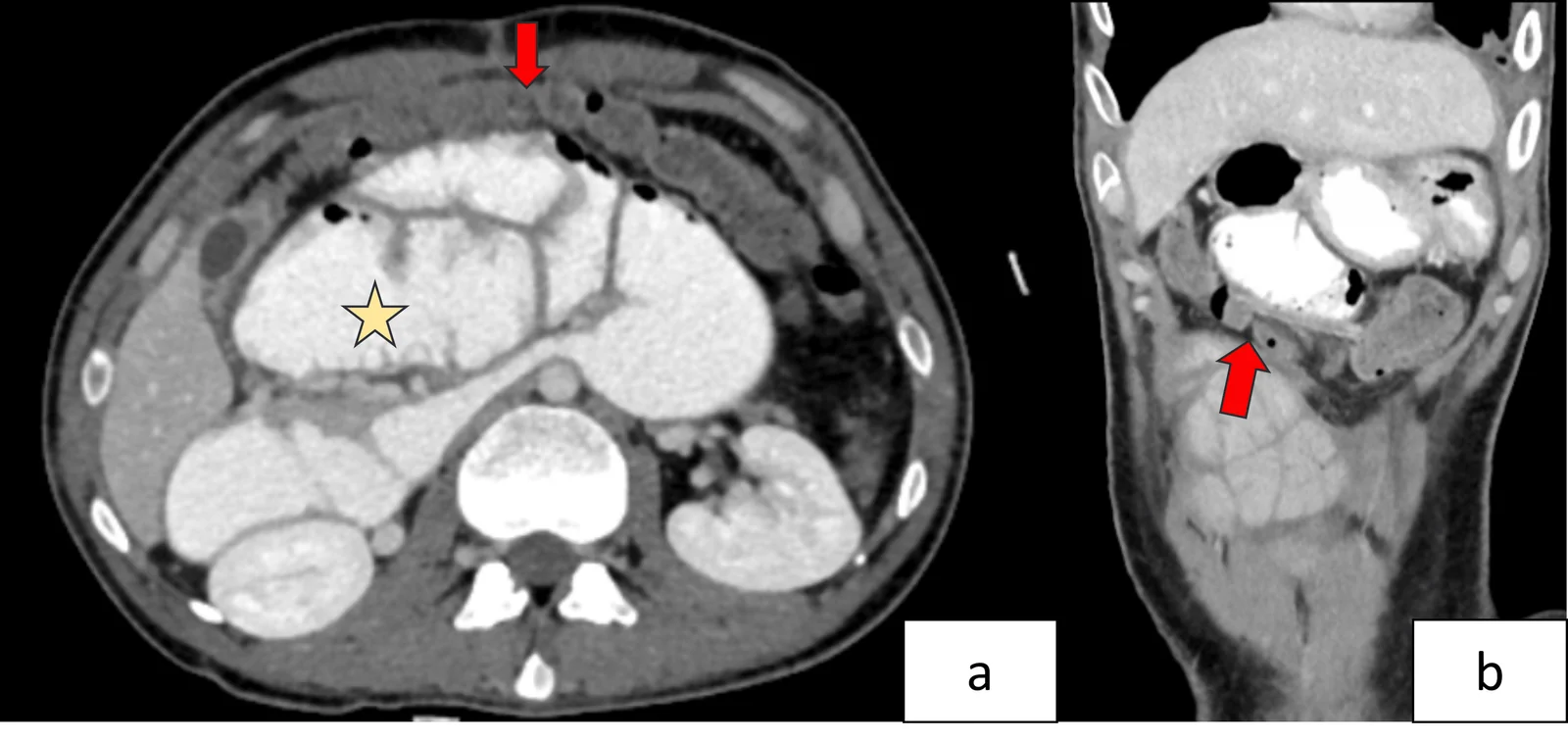
CECT images demonstrating transmesocolic internal hernia 3a Axial and 3b sagittal CECT images demonstrate dilated small bowel loops containing positive oral contrast within (yellow star) herniating through a transverse mesocolic defect. The herniated bowel loops are located posterior to the stomach and compress the transverse colon anteroinferiorly (red arrows), consistent with a transmesocolic internal hernia. CECT: contrast-enhanced computed tomography

**Figure 4:**
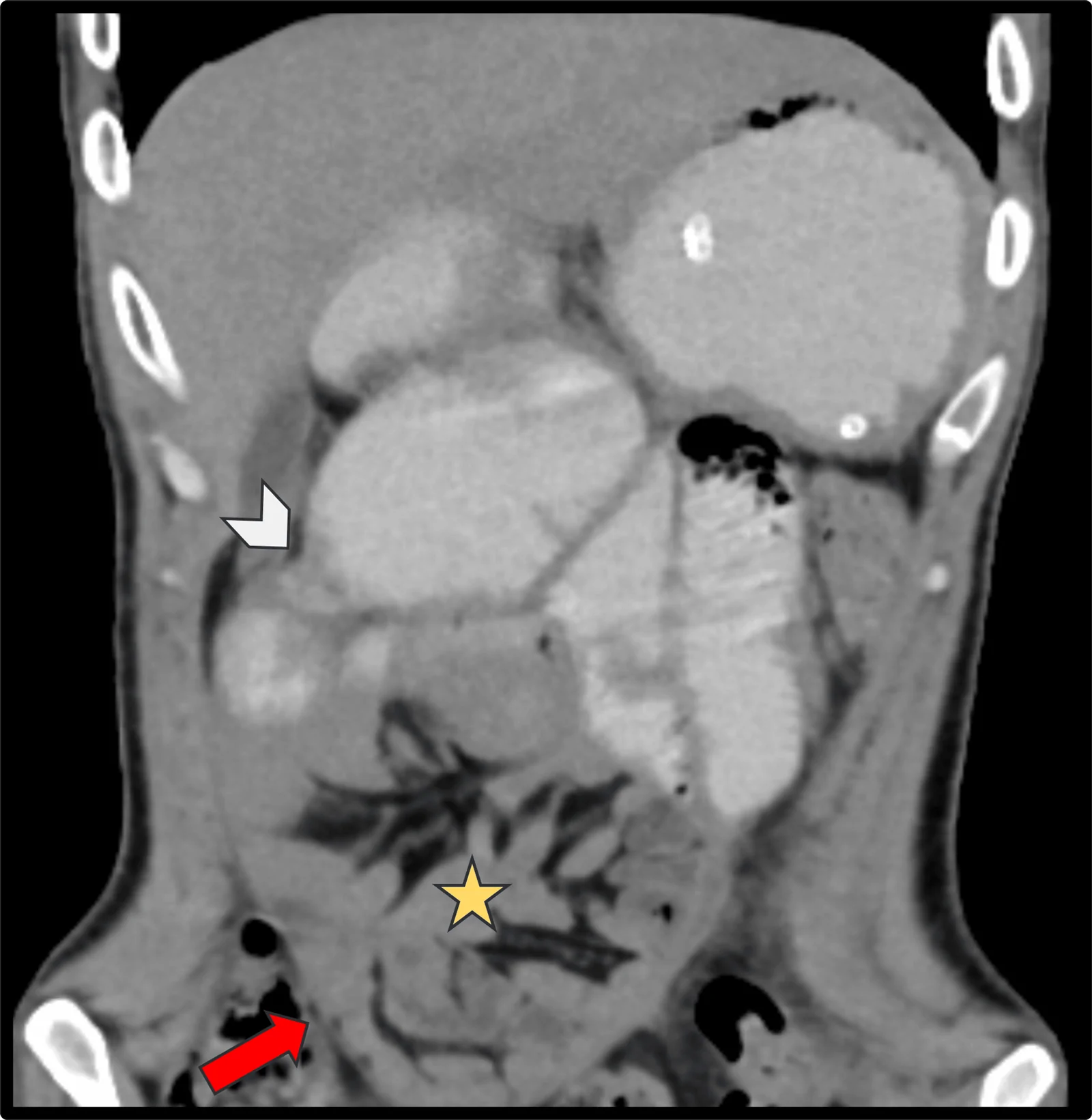
Coronal reconstructed CT image showing the transition point at the site of the transmesocolic internal hernia (white arrowhead). A separate focus of sclerosing EP is seen in the lower abdomen, with clustered small bowel loops (yellow star) enclosed by a fibro-collagenous membrane (red arrow), confirming the coexistence of two distinct pathologies EP: encapsulating peritonitis

Closer evaluation of the mesentery, however, revealed findings that were not entirely explained by EP alone. Dilated jejunal loops (maximum caliber 5.7cm) with abrupt convergence of mesenteric vessels through a focal defect within the transverse mesocolon, associated with a discrete transition point causing compression on the transverse colon, raised the possibility of a concomitant transmesocolic internal hernia (Figures 3, 4). Bowel wall enhancement was preserved throughout, with no CT evidence of pneumoperitoneum, closed-loop ischemia, or perforation.

**Figure 5:**
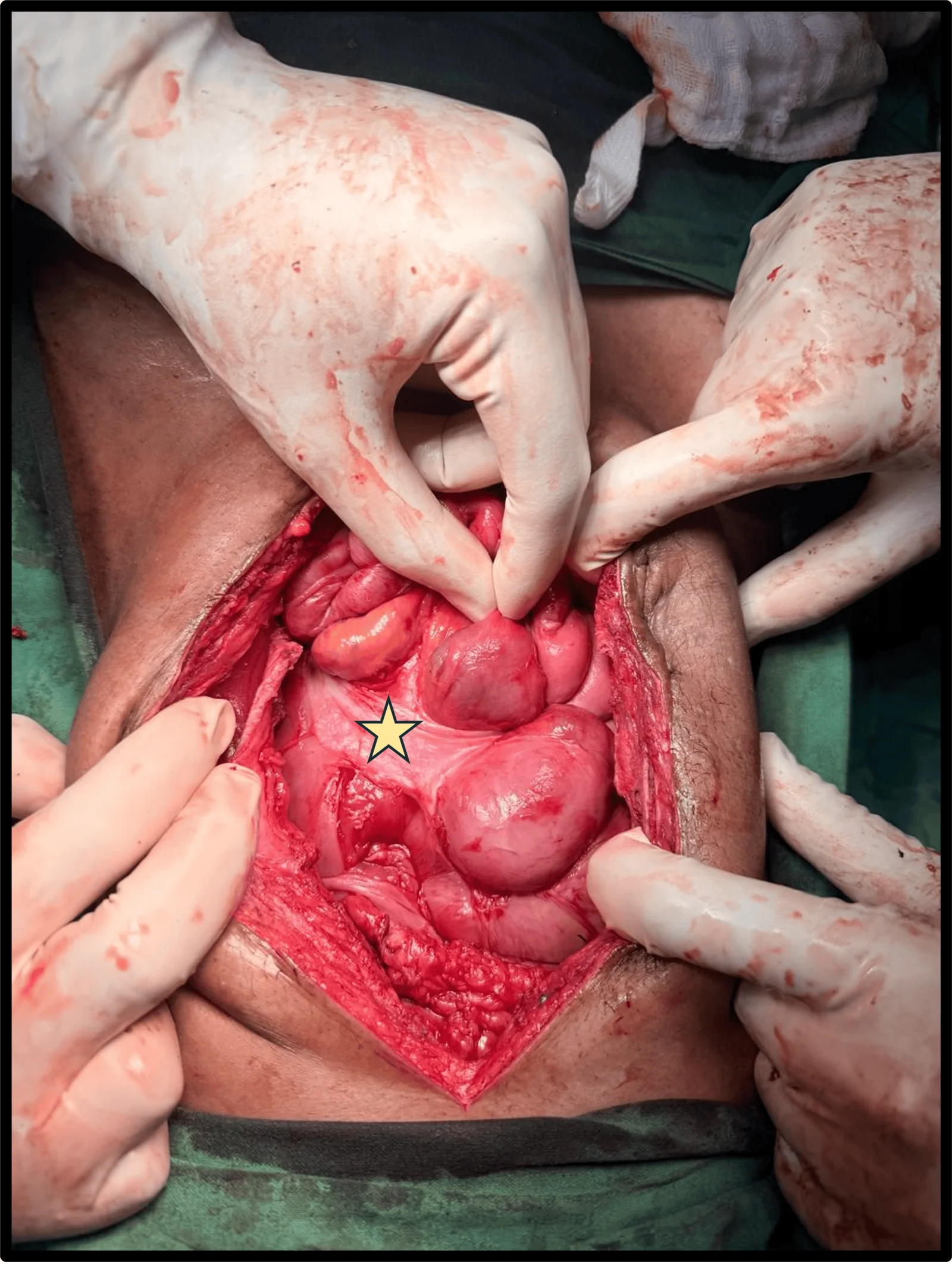
Intraoperative photograph demonstrating conglomerated small bowel loops enclosed within a fibro-collagenous cocoon, characteristic of EP (abdominal cocoon syndrome). The yellow star indicates the thick fibro-collagenous cocoon membrane encasing the bowel loops EP: encapsulating peritonitis

**Figure 6:**
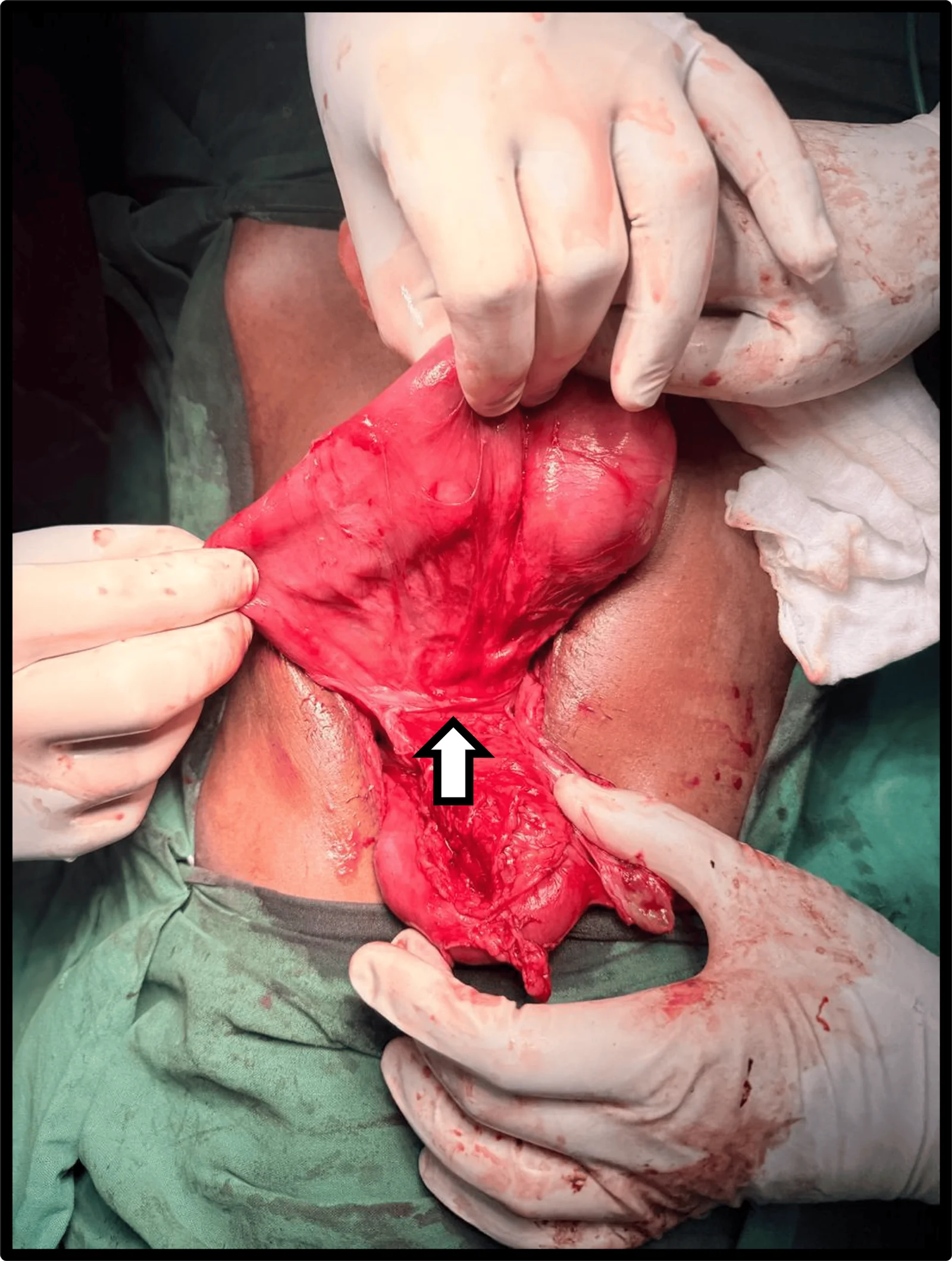
Intraoperative photograph demonstrating approximately 12 cm of herniated small bowel loops traversing a transverse mesocolic defect of 3 cm, consistent with a transmesocolic internal hernia. The white arrow indicates the transverse mesocolic defect through which the bowel has herniated

Given the persistent mechanical obstruction and the atypical combination of imaging findings, the patient underwent emergency exploratory laparotomy. Intraoperatively, the small bowel was found to be completely encased within a dense fibro-collagenous membrane, with extensive interloop adhesions producing the characteristic cocoon appearance (Figure 5). Following meticulous adhesiolysis and excision of the encapsulating membrane, further exploration unexpectedly identified a true defect of 3 cm within the transverse mesocolon containing approximately 12 cm of herniated small bowel loops, confirming the coexistence of a transmesocolic internal hernia (Figure 6).

The herniated bowel was viable and was reduced without the need for bowel resection. The mesocolic defect was subsequently closed using interrupted non-absorbable sutures.

The postoperative course was uneventful. Oral intake was resumed following the return of bowel function, and the patient was discharged in stable condition. At follow-up, he remained asymptomatic, with no evidence of recurrent intestinal obstruction.

## Discussion

Overview of sclerosing encapsulating peritonitis

Sclerosing EP, also known as abdominal cocoon syndrome, is an uncommon cause of SBO characterized by partial or complete encasement of the small bowel within a dense fibro-collagenous membrane. Once believed to predominantly affect adolescent females in tropical regions, it is now recognized across all age groups and both sexes. Based on etiology, EP is classified into primary (idiopathic) and secondary forms, the latter being associated with conditions such as long-term peritoneal dialysis, abdominal tuberculosis, recurrent peritonitis, previous abdominal surgery, ventriculoperitoneal shunts, autoimmune diseases, liver transplantation, intraperitoneal chemotherapy and other chronic inflammatory disorders. Despite increasing recognition, the diagnosis remains challenging because patients often present with nonspecific symptoms of intermittent or acute intestinal obstruction, and the condition is frequently identified only at surgery [1-5].

Internal hernias as a cause of small bowel obstruction

Internal hernias are another rare but clinically important cause of SBO and result from protrusion of bowel through congenital or acquired mesenteric or peritoneal defects. Although they account for only a small proportion of intestinal obstructions, delayed diagnosis may rapidly progress to closed-loop obstruction, strangulation, bowel ischemia, and perforation. Among the various subtypes, transmesocolic internal hernias are particularly uncommon and occur through defects in the transverse mesocolon [2,4].

Clinical and diagnostic overlap

The substantial overlap in the clinical presentation of EP and internal hernia poses a significant diagnostic challenge. Both entities may present with intermittent or acute abdominal pain, nausea, vomiting, abdominal distension, and features of bowel obstruction, while laboratory findings often remain nonspecific or unremarkable in the absence of ischemia. Consequently, contrast-enhanced CT plays a pivotal role in preoperative evaluation by accurately identifying the site, severity, and underlying cause of obstruction, while simultaneously assessing bowel viability [1-4,6,8].

Computed tomography findings and radiological differentiation

Although CT is highly sensitive for detecting bowel obstruction, distinguishing EP from an internal hernia can sometimes be challenging because both may demonstrate clustered bowel loops, a sac-like configuration, mesenteric crowding, proximal bowel dilatation, transition points, and interloop fluid. Nevertheless, several imaging findings aid differentiation. The defining feature of EP is a thick, enhancing fibro-collagenous membrane surrounding clumped bowel loops, often accompanied by interloop adhesions, peritoneal thickening, and the characteristic cauliflower or concertina appearance. In contrast, internal hernias are characterized by herniation of bowel through a mesenteric defect, with convergence of mesenteric vessels toward the hernial orifice, displacement of adjacent bowel loops, the mesenteric swirl sign, and, in advanced cases, features of closed-loop obstruction or bowel ischemia. Importantly, a true encapsulating membrane is typically absent in isolated internal hernias [2,4,5].

Comparison with previously reported cases

Arora et al. emphasized that internal hernia represents one of the closest radiological differentials of EP, particularly when bowel loops are clustered within a confined compartment. They highlighted that identification of an enhancing fibro-collagenous membrane strongly favors EP, whereas a true mesenteric defect is not expected. Similarly, Mansour et al. described a patient whose CT findings initially suggested an internal hernia but whose operative findings confirmed isolated EP, illustrating how the overlapping imaging appearances may lead to diagnostic uncertainty [5,6].

Distinctive features and novelty of the present case

Our case differs fundamentally from previously published reports because the two conditions did not merely mimic one another; they genuinely coexisted, which is rarely seen. Preoperative CT demonstrated imaging features characteristic of EP, including clustered bowel loops enclosed within an enhancing membrane, while simultaneously revealing mesenteric vascular convergence toward a transverse mesocolic defect and resultant dilated loops, raising suspicion for an associated internal hernia. Emergency laparotomy confirmed both pathologies, with dense fibro-collagenous encapsulation of the small bowel and a true transmesocolic internal hernia. To the best of our knowledge, reports documenting the coexistence of these two rare entities remain exceedingly scarce.

Notably, in our review of the available literature, we did not identify a previously reported case documenting this specific coexistence of EP with a transmesocolic internal hernia.

Radiological and surgical implications

The coexistence of EP and internal hernia carries important implications for both radiologists and surgeons. From a radiological perspective, recognition of an encapsulating membrane should not prematurely conclude the diagnostic evaluation. Careful assessment of the mesenteric vessels, transition point, and potential mesenteric defects remains essential, as concurrent pathology may alter surgical strategy.

Surgical management

The surgical management of EP and internal hernia is fundamentally distinct and is dictated by the underlying pathology. In EP, the primary surgical objective is complete excision of the encasing fibro-collagenous membrane with meticulous adhesiolysis, while preserving viable bowel and avoiding unnecessary resection. In contrast, management of an internal hernia involves reduction of the herniated bowel, careful assessment of bowel viability, resection of nonviable segments when indicated, and definitive closure of the mesenteric defect to prevent recurrence. In the present case, the coexistence of both entities necessitated simultaneous treatment of both pathological processes, comprising excision of the fibro-collagenous membrane with adhesiolysis, reduction of the herniated bowel, and closure of the transverse mesocolic defect [1-4,9,10].

This case reinforces an important diagnostic principle: radiological differential diagnoses are not always mutually exclusive. When clustered small bowel loops are encountered on CT, systematic evaluation of both the bowel and mesentery is essential, even after a seemingly definitive diagnosis has been established. Maintaining this broader diagnostic perspective may facilitate recognition of concurrent pathology, optimize operative planning, and ultimately improve patient outcomes.

## Conclusions

EP and transmesocolic internal hernia are uncommon causes of SBO that are traditionally regarded as radiological differentials because of their overlapping imaging appearances. This case demonstrates that these entities may coexist rather than merely mimic one another. Recognition of an encapsulating fibro-collagenous membrane should not preclude careful evaluation for mesenteric vascular abnormalities or defects suggestive of an associated internal hernia. A systematic assessment of both the bowel and mesentery on CT can facilitate accurate preoperative diagnosis, optimize surgical planning, and reduce the risk of overlooking concurrent pathology.
